# Bio-Packaging Based on Pectin/Tragacanth Gum with Added Extracts of Cherry Waste from the Wine Industry as a New Generation of Active Films for the Food Industry

**DOI:** 10.3390/foods14132203

**Published:** 2025-06-23

**Authors:** Renata Dobrucka, Lukas Vapenka, Marcin Szymański, Mikołaj Pawlik, Małgorzata Lasik-Kurdyś, Małgorzata Gumienna

**Affiliations:** 1 Department of Non-Food Products Quality and Packaging Development, Institute of Quality Science, Poznan University of Economics and Business, al. Niepodległości 10, 61-875 Poznan, Poland; mikoajpawlik5@gmail.com; 2 Department Food Preservation, University of Chemistry and Technology Prague, Technická 3, 166 28 Budějovice, Czech Republic; lukas.vapenka@vscht.cz; 3 Center for Advanced Technologies, Adam Mickiewicz University in Poznan, ul. Uniwersytetu Poznańskiego 10, 61-614 Poznan, Poland; marcin.szymanski@amu.edu.pl; 4 Department of Food Technology of Plant Origin, Faculty of Food Science and Nutrition, Poznan University of Life Sciences, ul. Wojska Polskiego 31, 60-624 Poznan, Poland; malgorzata.lasik@up.poznan.pl (M.L.-K.); malgorzata.gumienna@up.poznan.pl (M.G.)

**Keywords:** active materials, antioxidant packaging material, extracts from fruit wine pomace

## Abstract

In the present paper, extracts from pomace after cherry wine production were used as biocomponents of antioxidant packages. In the study, the highest concentrations of polyphenolic compounds were obtained when a 50% ethanol solution was used as the extraction solution. The addition of extracts provided statistically significant (*p* < 0.05) changes in water vapor transmission for the films obtained. The WVTR results are at a very low level, as values ranging from 7.96 ± 0.33 [g/m^2^ d] (sample 2) to 10.95 ± 0.33 [g/m^2^ d] (sample 1) were obtained. The addition of extract also affected the oxygen barrier. Samples without extract addition showed an OTR value of 2.42 ± 0.23 [cm^3^/m^2^ d 0.1 MPa]. A statistically significant (*p* < 0.05) reduction in this parameter was affected by the addition of extract to the matrix. Oxygen barrier properties ranged from 0.50 ± 0.05 (sample 3) to 0.94 ± 0.04 (sample 1), indicating high barrier properties of the packaging material. The addition of extracts caused an increase in opacity: films 3 and 4 were characterized by the highest value of the parameter, which was, respectively: 18.14 ± 27.02 and 18.97 ± 29.83 [%]. The research carried out in this study allows us to conclude that bioactive films with high application potential have been achieved and, in addition, represent a natural and ecological alternative to the materials currently used.

## 1. Introduction

Most of the current food packaging is made of synthetic materials derived from non-renewable sources. Although these materials possess good barrier and mechanical properties, their use is associated with the emission of significant amounts of persistent, non-biodegradable solid waste, posing a significant environmental challenge. Due to its protective function and importance in maintaining food quality, packaging is indispensable at all stages of food product production, storage, and distribution [1,2,3]. Growing environmental awareness among consumers and their increasing requirements are driving the development of the packaging market and intensifying investments in sustainable packaging solutions [1]. According to the latest forecasts, the global sustainable packaging market is expected to grow at a rate of 7.7% per year, reaching a value of USD 470.3 billion by 2027. This dynamic is due to both consumer pressure and legal regulations promoting more environmentally friendly alternatives. The growing trend of replacing conventional plastics with biopolymers for packaging is driven by increasing environmental concerns and demand for materials with a more favorable environmental profile. Biopolymers are of particular interest from the perspective of sustainable development and consumer safety, as their biodegradable, non-toxic, and biocompatible properties make them a viable alternative to traditional plastics. Currently, bio-based packaging made from natural polymers accounts for around 1% of the packaging market in Europe. The low representative proportion of these materials is due to, among other things, high production costs and difficulties in ensuring stable and sustainable sources of bio-based raw materials [2,3]. However, regulatory pressure, changing consumer preferences, and technological progress in the field of materials science are contributing to the growing importance of this market segment and are intensifying efforts to develop innovative, more sustainable solutions [1,3]. As a result, the packaging sector is currently undergoing a phase of gradual transformation, in which the industry and related supply chains are adapting to new environmental and system requirements, which are heading towards more ecological and responsible packaging strategies.

The scale of food loss and waste has reached a level that qualifies this phenomenon as a global challenge with significant economic, social, and environmental consequences, covering all stages of the agri-food chain [4,5,6,7]. It is estimated that about one-third of food intended for human consumption is wasted, mainly in the form of residues from processing, which translates into about 1.3 billion tons per year [4]. Such significant amounts of food waste also contribute to environmental degradation, because in anaerobic decomposition conditions in landfills, they emit toxic greenhouse gases. In countries such as China, India, the Philippines, and highly developed regions of the United States, the annual production of waste generated during the processing, packaging, distribution, and consumption of fruit and vegetables reaches about 55 million tons [5]. In this context, issues of quality and safety of packaged food are of particular importance [6]. Among innovative solutions in the field of food packaging technology, packaging with antioxidant properties has been indicated as one of the most promising tools enabling a significant delay of oxidation processes [7]. Antioxidants contained in packaging materials can capture free radicals and chelate metal ions, which limit oxidative reactions and extending the shelf life of food products. Currently, synthetic commercial antioxidants dominate in practical applications, but their use raises concerns due to potential toxicity and limited usefulness in the context of contact with food [1,8]. An alternative are antioxidants of natural origin, which are considered safer and may additionally have a beneficial effect due to the presence of bioactive compounds.

Post-production waste from fruit and vegetable processing is a rich source of natural antioxidants with high biological potential. It is mainly a result of processes such as juicing and the production of wine, jams, jellies, marmalades, mousses, or fruit cocktails. Pomace––a mixture of pulp, peels, seeds, and stems––often contains much higher concentrations of bioactive compounds than the liquid product itself, e.g., juice [9,10,11,12]. However, the management of these residues poses a significant technological and logistical challenge for the food industry, especially due to their seasonal nature and rapidly appearing surpluses in a short period of time. Traditional applications include use as organic fertilizers, although the presence of high concentrations of polyphenols can inhibit plant growth, and as feed, where the lignins they contain, phenolic polymers, reduce digestibility [13]. Plant by-products resulting from food processing constitute a currently-underutilized fraction of biomass, rich in numerous bioactive compounds of a functional nature. These include, among others, phenolic compounds, proteins, dietary fiber, polysaccharides, flavoring substances, and other phytochemical compounds. They are characterized by a wide spectrum of biological activity, including antioxidant, antimicrobial, and prebiotic properties [14]. Therefore, effective management of plant by-products is consistent with the concept of sustainable development within the food sector and is an important element in achieving global goals regarding sustainable production and consumption models [11].

According to the current state of knowledge, there are only a limited number of reports in the available world literature on the practical use of industrial waste as sources of active substances for the production of packaging materials [1,2,3,7,8,11]. Few studies describe the use of polysaccharides, such as apple pectin and gum tragacanth, in the context of developing innovative packaging materials. Both of those biopolymers have been classified by the Joint FAO/WHO Expert Committee on Food Additives (JECFA) as food additives with a safe toxicological profile [5]. Pectin, as an anionic polysaccharide with biodegradable, biocompatible, non-toxic, and edible properties, can bind cationic active substances, which allows the formation of membranes and coatings characterized by favorable mechanical and barrier properties [15]. On an industrial scale, it is obtained, among other sources, from apple pomace––a by-product of fruit processing. In addition to its traditional applications as a gelling, thickening, and stabilizing agent, pectin is increasingly used as a functional ingredient with health-promoting effects, including as a fat substitute in food products [16]. Gum tragacanth is a natural biopolymer obtained mainly from the exudate of plants of the Astragalus genus, especially Astragalus gummifer, occurring mainly in the Middle East. It is a complex mixture of highly branched, heterogeneous polysaccharides, the dominant fraction of which is tragacanth acid, a polymer composed of α-D-galacturonic acid units connected by α-1,4 bonds. The presence of numerous carboxyl, hydroxyl, and methoxyl groups gives the gum ionic properties, enabling interaction with metal ions and polymers of the opposite charge. In addition, it is characterized by good miscibility with other gums and many biopolymers, which makes it a valuable ingredient in the design of functional polymeric materials [17]. In this study, pomace extracts were used as antioxidant biocomponents to develop packaging materials based on apple pectin and gum tragacanth. The aim was to obtain functional packaging materials with antioxidant properties and potential for applications in sustainable food packaging technologies.

## 2. Materials and Methods

### 2.1. Materials

Wine pomace, after the production of cherry wine with the addition of green tea infusion, was applied in the performed experiment. The wine was produced in the Fermentation and Biosynthesis Laboratory at the University of Life Sciences in Poznan, Poland. The process has been described in detail in the literature [18]. After seven days of cherry pulp maceration, pomace was obtained, then frozen and stored at −20 °C. Before extraction, the pomace was freeze-dried (main drying: 19 h; 0.12 mbar, temp. −40 °C, post-drying: 5 h; 0.1 mbar; temp. −42 °C).

### 2.2. Extracts Preparation

The freeze-dried pomace was crushed in a mortar to obtain a powder, then 5 g of pomace powder and 150 mL of solvent (water or 50% *v*/*v* ethanol solution (ethanol, Sigma-Aldrich, Munich, Germany)) were placed in a 250 mL Erlenmeyer flask and sealed tightly with a stopper (assumption: 30 mL of extraction solvent per 1 g of pomace powder). Table 1 presents the extraction variants.

The applied ultrasonic bath used (Polsonic Palczynski, Poland) operated with a power of 80 W. The extracts were filtered through paper filters and then concentrated using a vacuum evaporator (Buchi, Flawil, Switzerland, vacuum pump V-700, vacuum controller V-850, rotavapor R-215). Water extracts were concentrated using a pressure of 100 mbar, 60 rpm, bath temp 60 °C, vapor temp 45 °C, time about 20 min, while ethanol extracts were concentrated using a pressure of 130 mbar, 60 rpm, bath temp 50 °C, vapor temp 38–40 °C, time about 20 min.

### 2.3. Preparation of Films

Extracts prepared previously were combined with a % apple pectin (PA) solution containing glycerol (0.5% *w/w*) and tragacanth gum (TG) in a ratio of 1:1:1. The solutions were put under magnetic stirring at 500 rpm for 95 min at 120 °C. The extracts were added to the prepared solution in a ratio of 1:10. The obtained solutions were put under magnetic stirring at 600 rpm for 45 min at 120 °C. Each film-forming solution was dried at 25 °C for 72 h in order to produce a thin film.

### 2.4. Evaluation of the Bioactive Potential of Extracts and Films

The extracts for analysis were prepared in appropriate dilutions. In the case of films, squares of 1 × 1 cm were cut out and weighed. Then the squares of films were placed in the solvent in order to elute polyphenols from the film surface. Next, after 30 min., the concentration of polyphenols in the solutions was tested.

### 2.5. Total Polyphenols Content

The total polyphenol content was measured by the Folin–Ciocalteau method [19] with our modifications [20]. Specifically, 0.3 mL of extract solution was mixed with 4.15 mL deionized water, 0.05 mL of Folin–Ciocalteu reagent (Sigma-Aldrich, Munich, Germany), and 0.5 mL of 5% 2 M sodium decarbonate solution (POCh, Gliwice, Poland). After mixing, the samples were incubated at room temperature for 20 min. After incubation, the absorbance of the samples was determined at 700 nm. The data were calculated as chlorogenic acid equivalent per 1 g of freeze-dried pomace (g CAE/g; Sigma-Aldrich, Munich, Germany). In the case of the determination of total polyphenols in the tested films, the results were expressed as mg CAE/g of the tested films.

### 2.6. Antioxidant Activity

The antioxidative capacity (TEAC) was determined against the ABTS reagent (2,20-azinobis-(3-ethylbenzothiazoline-6-sulphonic acid) (Sigma-Aldrich, Munich, Germany) according to the method described by Re et al. (1965) in our modification. The analysis was performed as follows: 3 mL of ABTS reagent (Sigma-Aldrich, Munich, Germany) and 30 µL of extract solution were added to and tightly sealed within a glass tube, mixed, and incubated. Next, spectrophotometric measurement was performed at 735 nm (Halo SB-10 Dynamica Biogenet, Cambridge, UK). Results were expressed as the capability of antioxidants to scavenge ABTS radicals, relative to that of Trolox (Sigma-Aldrich, Munich, Germany), and were presented as mg of Trolox (a water-soluble vitamin E analogue) equivalent per 1 g of freeze-dried pomace (mg TE/g). In the case of the determination of the antioxidant activity of the tested films, the results were expressed as mg TE/g of film [21].

### 2.7. FTIR Study

The FTIR analysis was performed using a Thermo Scientific Nicolet iS50 FTIR spectrometer (Thermo Fisher Scientific, Waltham, MA, USA).

### 2.8. Barrier Properties of Active Eco-Packaging Materials

Water vapor transmission rate (WVTR) was determined by the gravimetric method according to standard [22] at 23 °C and relative humidity of 50%. Permeance of the film to oxygen gas was determined using OxTran 2/20 MH measuring system (MOCON, 7500 Mendelssohn Ave. N Minneapolis, MN, USA) according to standard [23] at 23 °C and relative humidity of 50%. An aluminum self-adhesive mask for reduction of sample tested area (5.73 cm^2^ instead of the standard 50.00 cm^2^) was used for permeance. Due to the different thicknesses of the films, the transmittance values were referred to a uniform thickness of 100 μm.

### 2.9. Migration Test

**Figure 1:**
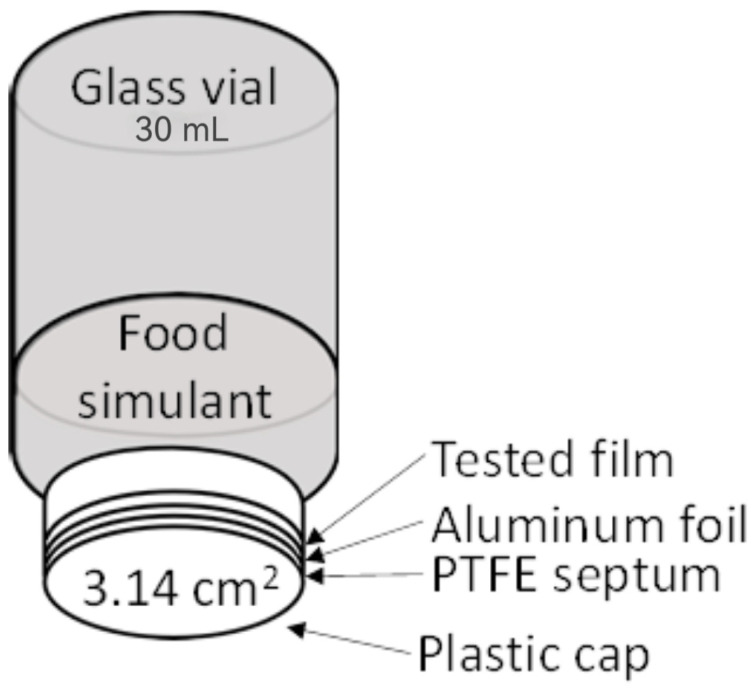
Arrangement of a migration test in a glass vial.

Migration tests were performed in principle according to the series of standards [24]. Testing conditions of 10 days at 40 °C were performed according to the requirements of Regulation 10/2011 for covering storage at room temperature or below, including when packaged under hot-fill conditions, and/or heating up to a temperature of 100 °C for a maximum time of 15 min. Overall migration into neutral food simulant–10% ethanol (*w*/*w*), into evaporable substitution of fatty food simulant–95% ethanol (*w*/*w*) and dry food simulant–modified polyphenylene oxide (MPPO, Tenax^®^ TA, 60-80 mesh) (Thermo Fisher Scientific, MA, USA) was tested (Figure 1). The tests were carried out in 30 mL glass vials equipped with a plastic screw cap with a Teflon septum and an aluminum foil insert. A foil sample with a total area of 3.14 cm^2^ was placed in the lid (see Figure 1) in the amounts of 3.75 mL (10% or 95% ethanol) or 0.12 g (MPPO). Subsequently, the vial was sealed and turned bottom up. Under the given period for migration test, the level of overall migration was determined gravimetrically for 10% ethanol and 95% ethanol by weighing of non-volatile residue on microbalance XPR36 (Mettler Toledo, Switzerland) after evaporation of simulant from the vials and used for migration test at 90 °C for 10% ethanol and at 75 °C for 95% ethanol in drying chamber with natural convection. Determination of overall migration into dry food simulant was processed by three-step extraction of Tenax^®^ by shaking (1 min) by diethyl ether (1st 2.4 mL, 2nd, and 3rd 3.6 mL) in a glass vial used for migration test, with decanting and collecting of each diethyl ether extract through a filter into a new 15 mL glass vial. Non-volatile residue for microbalance weighing was obtained by evaporating diethyl ether from the filtered collected extracts in a 15 mL vial on a dry bath sample concentrator MINI-100N (Miulab, Hangzhou City, China) at a temperature of 30 °C and constant flow of nitrogen above the diethyl ether extracts level. All laboratory consumables and chemicals used above were purchased from Sigma-Aldrich (Merck, Darmstadt, Germany) and P-LAB (Prague, Czech Republic).

### 2.10. Mechanical Properties

Strength testing (elongation at break [EB] and tensile strength [TS]) was carried out on a Zwick model BDO-FBO 0.5TH testing machine. The test was carried out similarly to the test carried out in references [6,25].

### 2.11. Opacity Research

Transmittance, transparency, and haze tests were carried out on a Eurotom Bull haze gard I machine. Testing was carried out by ASTM D1003-13. The percentage transmittance, transparency, and haze exhibited by the samples were examined.

### 2.12. Images Analysis

Microscopy studies were performed using an Evo 40 scanning electron microscope (Zeiss, Oberkochen, Germany) operating in beam mode at 20 kV with a secondary electron detector. A stereoscopic Zeiss SteREO Discovery V8 microscope (Zeiss, Oberkochen, Germany) was also used.

### 2.13. Statistical Analysis

#### 2.13.1. Spearman Rank Correlation

For correlation verification between the study samples, parameter values (Transmittance, Haze, Transparency, Tensile strength, WVTR, Total polyphenols, antioxidants, migration, oxygen permeability) were collated, and Spearman’s rank correlation test was performed. Correlation coefficients are significant at *p* < 0.05. Calculations were performed using STATISTICA 13. Variables were assumed to be: not correlated R = 0; weakly correlated 0 < R < 0.5; highly correlated 0.5 ≤ R < 0.7; very highly correlated 0.7 ≤ R < 0.9; almost fully correlated 0.9 ≤ R < 1; fully correlated R = 1.

#### 2.13.2. Cluster Analysis

To illustrate the ‘data structure’ for the determined parameters, total polyphenols and antioxidant activity, all assay results were converted to standardized values according to the equation:

(x_i_ − x_m_)/SD (1)

where:

x_i_—value of a single result,

x_m_—mean value of a given parameter,

SD—standard deviation of the parameter

That transformation allows the results to be achieved in dimensionless form before proceeding to cluster analysis. The analysis involves several different classification algorithms and can be used to detect structures in data sets, without interpreting or explaining any relationships. Cluster analysis is not a statistical test: rather, it is a set of different algorithms that ‘order objects into clusters’. Such clustering is used in many research areas. If some data have a clear ‘structure’, i.e., there are clusters of objects that are similar to each other, this structure will most often be reflected in a hierarchical tree as separate branches. The agglomeration method allows clusters (branches) to be detected and interpreted. To create clusters, measures of divergence or distance between objects are used. The most common type of distance is the euclidean distance, which is the metric distance in a multidimensional space. It is calculated from the formula:

(x, y) = {Σ_I_ (x_i_ − y_i_)^2^}^½^ (2)

This was calculated using STATISTICA ver. 13.

## 3. Results and Discussion

### 3.1. Testing Antioxidant Activity and Total Polyphenols in Pomace Extracts After Cherry Wine Production and in Prepared Films

Extraction is an important element in the recovery of bioactive components from the plant matrix. Traditional techniques are often time-consuming and require relatively large amounts of solvents. Furthermore, without the selection of optimal parameters such as time, temperature, oxygen access, etc., high losses of polyphenols may occur through ionization, hydrolysis, or oxidation [11,13]. Bioactive compound recovery is strictly determined by the selection of an appropriate solvent as well as the sample-to-solvent ratio, especially considering the polar nature of the compounds [26,27]. The extraction conditions should be aimed at recovering the compounds of interest with the shortest possible extraction time, selecting solvents with the least environmental impact, and reducing the number of organic solvents and energy factors used [26,28,29,30]. Solvents such as methanol, ethanol, acetone, and water are mainly used to recover polyphenols from pomace after winemaking [27,31,32]. Analysing the extraction efficiency of total polyphenol content (TPC), ethanol/water mixtures showed relatively better results compared to acetone/water or methanol/water mixtures [12]. Other major benefits worth underlining are that ethanol is cheaper and has GRAS (Generally Recognized As Safe) status (as defined by the US Food and Drug Administration). It is therefore far less controversial compared to methanol or acetone as a solvent generally used in the food industry. In the present study, water and 50% ethanol were tested as extraction solvents. The ethanol solution was chosen because the polar nature of polyphenols indicates that they dissolve readily in polar solutions, such as water–alcohol solutions. In studies on optimizing extraction parameters for polyphenolic compounds from cherry pomace, researchers indicate that 50% ethanol was a very effective extraction agent [26,27]. In addition, many studies have shown that the use of water–alcohol solutions is beneficial because the presence of water increases the permeability of the cellular tissue, allowing better mass transfer by molecular diffusion [32,33].

The prepared extracts were evaluated for their content of polyphenolic compounds and antioxidant activity. Conventional solid–liquid extraction was modified with ultrasound-assisted extraction. The application of this technique was aimed at breaking down the cell structure in the pomace biomass (mainly the peels) to increase the extraction of bioactive compounds. Similarly, ultrasound-assisted extraction has been used by other authors to recover bioactive compounds from various agri-food wastes and by-products [9,28,34,35,36,37].

**Figure 2:**
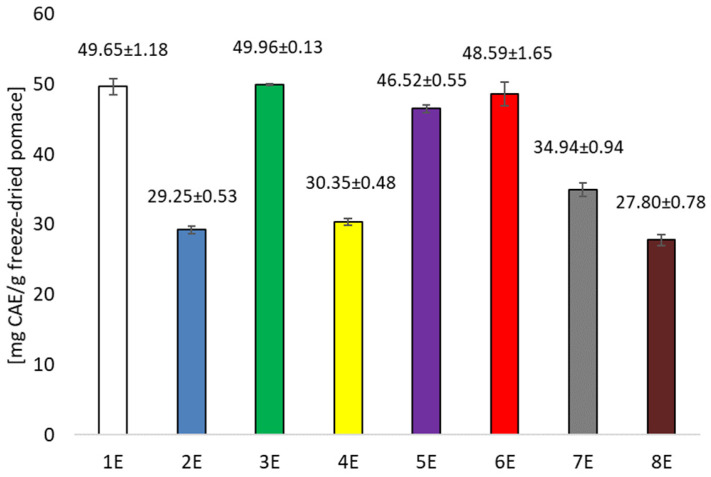
Concentration of total polyphenols in the extracts (calculated as mg of chlorogenic acid in 1 g freeze-dried pomace).

In the present study, by far the highest concentrations of polyphenolic compounds were achieved when a 50% ethanol solution was used as the extraction solution. The best option, in terms of polyphenol extraction efficiency, was 30 min of sonification using 50% ethanol. An increase in the sonification process time to 2 h did not increase the extraction efficiency, which is a very important observation from the point of view of process economics. Extraction with ethanol solution under shaking conditions yielded similar polyphenol yields to sonification (Figure 2), and no significant effect of increased temperature on the change in extraction efficiency was shown. Water extracts had a significantly lower concentration of polyphenolic compounds.

**Figure 3:**
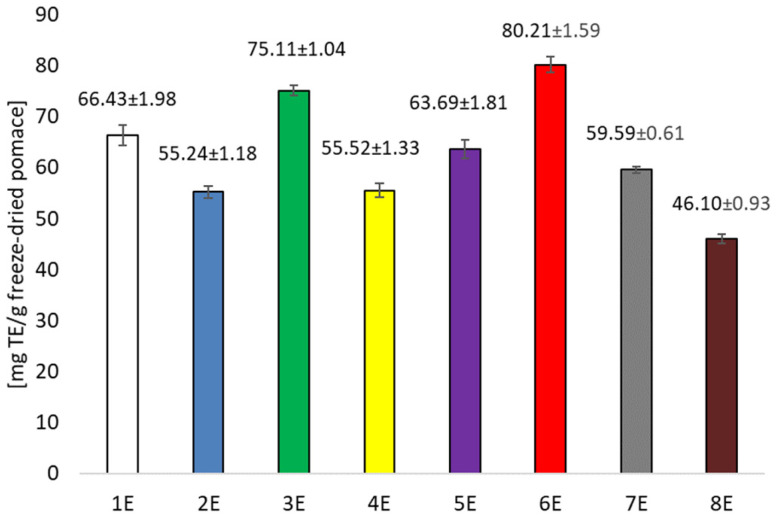
Antioxidant capacity TEAC in the obtained extracts (calculated as mg of Trolox equivalent in 1 g freeze-dried pomace).

**Figure 4:**
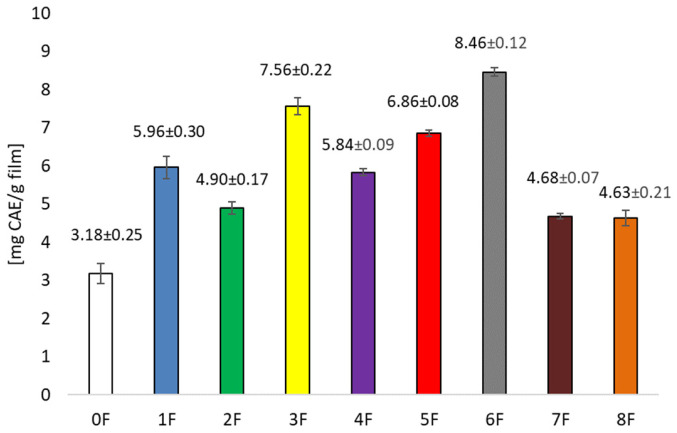
Concentration of polyphenols in the films (calculated as mg chlorogenic acid in 1 g of film).

**Figure 5:**
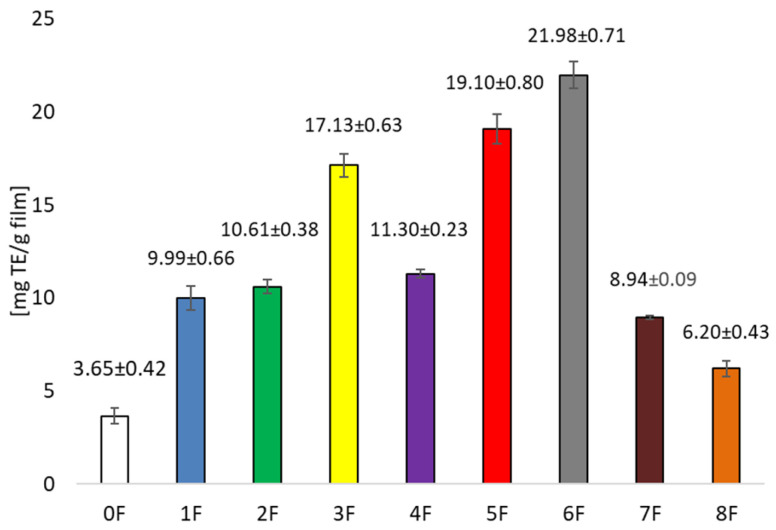
Antioxidant capacity TEAC in the obtained films (calculated as mg of Trolox equivalent in 1 g of film).

Analysis of the antioxidant potential of the extracts obtained also showed significantly higher values for the ethanolic extracts compared to the aqueous extracts. However, greater variation was noted. Prolonging sonification from 30 to 120 min increased the antioxidant potential of the extract significantly (Figure 3). In contrast, under stirred conditions, increasing the temperature from 25 to 60 °C had a negative effect on the antioxidant activity of the extracts tested. This indicates that qualitative and quantitative changes in the profile of polyphenolic compounds occur under the influence of the extraction variants used (sonification, temperature, stirring). The extracts were used to produce bioactive organic films, which were also evaluated for their polyphenolic compound content and antioxidant activity. Films containing ethanol extracts showed significantly higher amounts of polyphenolic compounds compared to films with aqueous extracts, with the film containing the extract gained by shaking at 60 °C having the highest content (Figure 4). Significantly lower values were recorded in films containing aqueous extracts. Similar relationships were noted when the antioxidant potential of the films was tested. The highest activity was recorded for films with ethanol extract after shaking at 60 °C (Figure 5), while significantly lower when the extracting agent was water. Examining the influence of the factors tested—type of extracting solvent, temperature and extraction technique—it was observed that the type of extracting agent had the greatest influence on extraction efficiency. The highest extraction efficiency of polyphenols in cherry pomace after winemaking was achieved using a 50% ethanol solution.

### 3.2. FTIR Study of Eco-Friendly Packaging Materials

**Figure 6:**
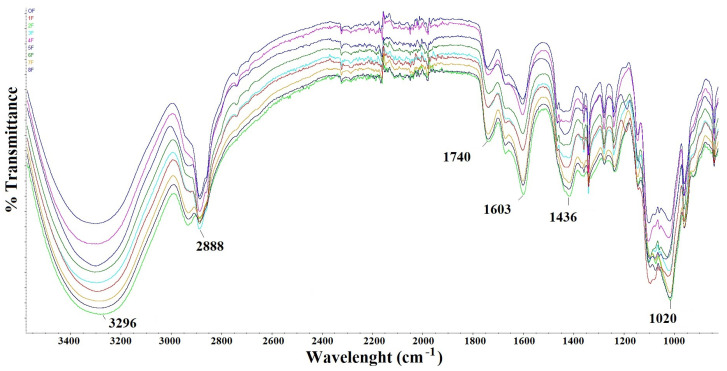
FTIR spectra of eco-friendly packaging materials.

On the spectrum (Figure 6), the peaks are at the values 3296 cm^−1^, 2888 cm^−1^, 1740 cm^−1^, 1603 cm^−1^, 1436 cm^−1^, and 1020 cm^−1^. The broad and intense bands at 3296 cm^−1^ and the weak bands around 2888 cm^−1^ are attributed to O–H stretching vibrations of the hydroxyl group and C–H stretching vibrations [38]. The stretched bands for all films are characteristic of O–H stretching, which corresponds to the axial deformation of the hydroxyl–O–H found in pectic structures, but may also be related to O–H vibrations of water molecules in the structure of the resulting films [39]. It also indicates the presence of hydrogen bonds between pectin, gum tragacanth, and extract. This also demonstrates the stable structure of the films [6]. The prominent peaks at 1740 cm^−1^ and 1603 cm^−1^ were from protonated carboxylic acid (COOH) and amide I (C–O stretching) [38]. The band at 1020 cm^−1^ is attributed to C–O–C elongation vibrations of the polymer chain structure [40]. Nevertheless, the usable spectra confirm the stable study of the prepared films.

### 3.3. Barrier Research of Eco-Friendly Packaging Materials

This study tested the barrier properties (water vapor transmission rate and oxygen permeability) of the received eco-friendly packaging materials. Both of these parameters have an impact on the quality of the packaging material and whether it is permeable to oxygen or water vapor. The storage life of both food and non-food products is directly linked to the permeation of water vapor between the product and the external environment. One important function of food packaging is to minimize the permeation of water vapor and oxygen [41]. The pure pectin films were characterized by a WVTR value of 18.13 ± 1.16 [g/m^2^ d]. The addition of extracts provided statistically significant (*p* < 0.05) changes in water vapor transmission for the prepared films. It was observed that there was a decrease in the value of the WVTR parameter for all films, regardless of the type of extract used. Therefore, the addition of extract increased the barrier properties of the films. The relatively low WVTR value is beneficial for reducing the risk of microbial growth and maintaining food freshness [42].

**Figure 7:**
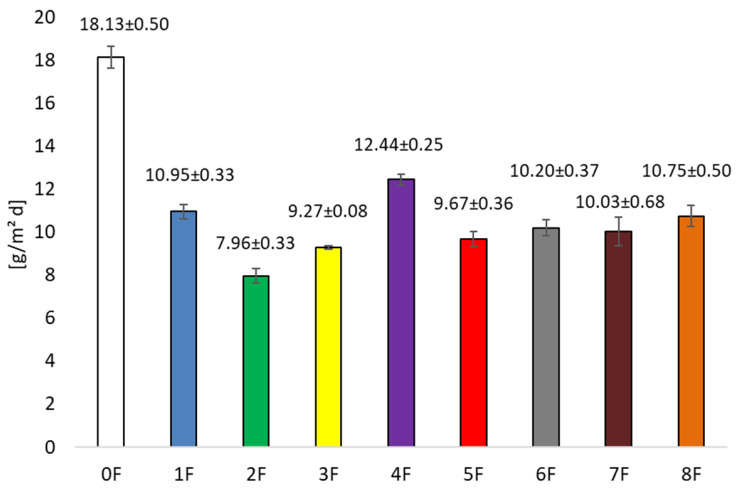
Water vapor transmission rate of tested films at conditions 23 °C, 50% relative humidity.

The WVTR results achieved in this study (Figure 7) were at a very low level, as values between 7.96 ± 0.33 [g/m^2^ d] (F2) and 10.95 ± 0.33 [g/m^2^ d] (F1) were obtained. There are many factors that affect the barrier properties of films, such as cross-link microstructure, water sensitivity, crystallinity, and the ratio of hydrophobic and hydrophilic components. In our case, the changes in water vapor transmission of the tested films were associated with the use of rich in active extracts. The reduction in WVTR can be attributed to strong intermolecular interactions between the excess phenolic compounds and the carboxyl/hydroxyl groups in the pectic chains, which hinder water binding by the pectic chains [43].

**Figure 8:**
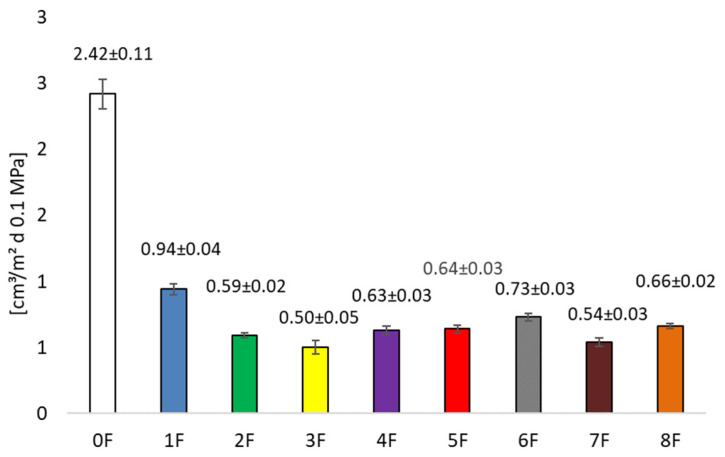
Permeance of the film to oxygen at conditions 23 °C, 50% relative humidity.

The addition of extract also affected the oxygen barrier. Figure 8 shows the permeance of the film to oxygen at conditions of 23 °C, 50% relative humidity. This parameter is of great importance for the preservation of the quality of the packaged product. Samples without extract showed values of 2.42 ± 0.23 [cm^3^(STP)/m^2^ d 0.1 MPa]. A statistically significant (*p* < 0.05) decrease in this parameter was influenced by the addition of extract to the matrix. Oxygen barrier properties ranged from 0.50 ± 0.05 (3F) to 0.94 ± 0.04 [cm^3^(STP)/m^2^ d 0.1 MPa] (1F). There is a very high barrier property of the packaging material, especially for polysaccharide films, in humid conditions [38]. Commonly used food packaging films with the same thickness, such as PP or LDPE, have higher values. The values for the films are evidence of the materials’ exceptionally high barrier to oxygen. The gas permeability of packaging materials is related to the crystallinity, degree of cross-linking, and segmental movement of the polymer chain in the polymer matrix. The results obtained in this study can be attributed to the appropriate compatibility and interaction between the film components and the formation of an ordered and compact network structure, which contributes to the high barrier properties towards the tested gases [38].

Essential was undoubtedly the incorporation of extracts (irrespective of how they were obtained) into the polymer matrix. Films with such a high barrier to gases are now a requested packaging material. The current trend is towards bio-monomaterials, preferably without metallization or blending with other materials. Thus, the solution proposed in this paper has great potential for use in a closed-loop economy. Statistical testing of Spearman’s rank correlation coefficients showed that WVTR is highly correlated with OTR (R = 0.7167) and highly correlated with migration in Tenax.

### 3.4. Mechanical Tests of Eco-Friendly Packaging Materials

In the present study, the mechanical parameters of the obtained pectin eco-friendly packaging materials were evaluated, as the mechanical strength of the films is essential to maintain the packaging integrity of food during storage, distribution, and transport. The mechanical properties of the films are therefore of great importance, considering the practical application of the films [44]. In this study, elongation at break (EB, %) was determined, which measures ductility, i.e., the ability of the film to stretch before breaking, while tensile strength (TS, MPa) measures the maximum stress the material sample can withstand when stretched before breaking.

**Figure 9:**
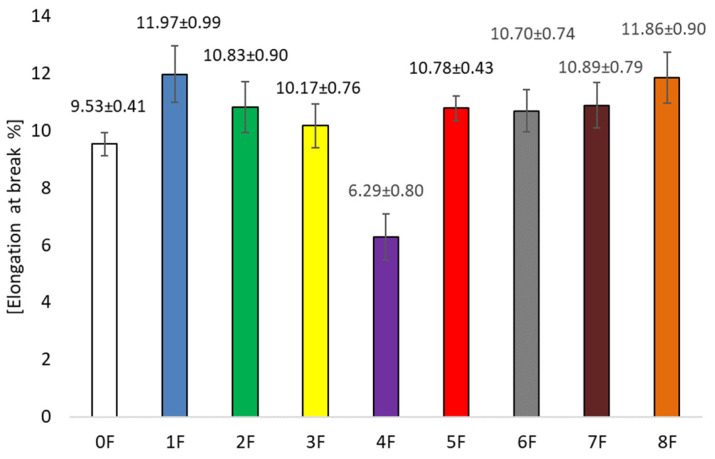
EB tests for the evaluated films.

**Figure 10:**
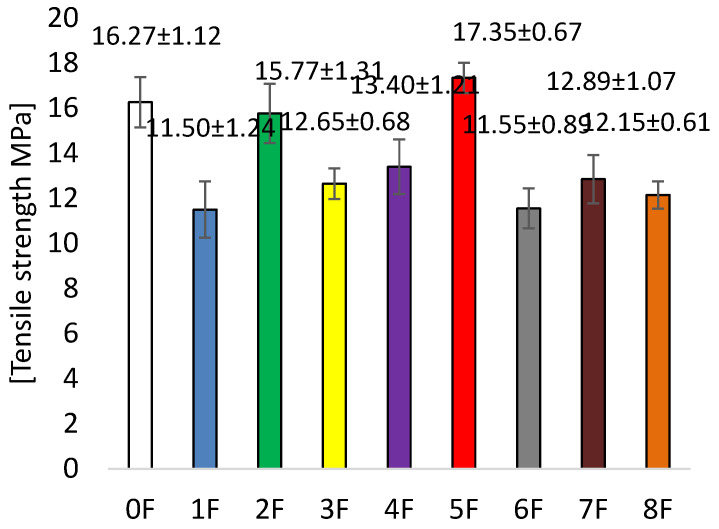
TS tests for the examined films.

For the samples obtained, an increase in flexibility was observed compared to the zero sample. Sample 0 was characterized by an EB value of 9.53 ± 0.41 [%] (Figure 9). The addition of extract increased the value of elongation at break, and its value increased depending on the preparation of the extract. For sample 4F only, the EB value was 6.29 ± 1.39 [%]. This is the result of a number of factors. These included the use of a specific extract, which was characterized by varying amounts of polyphenols and their interaction with pectin, plasticizer, and tragacanth gum. The result was a reduction in the attraction of intermolecular bonds in the three-dimensional structure of the polymer. The extracts used increased the polymer matrix’s free volume and chain mobility. Figure 10 shows the TS results for the tested films. It was observed that high TS values were obtained for sample 0 and sample 5, where values were at 16.27 ± 1.12 [MPa] (0F) and 17.35 ± 0.67 [MPa] (5F). The lowest TS value was gained for samples 1F (11.50 ± 1.24 [MPa]) and 6F (11.55 ± 0.89 [MPa]). Thus, the addition of extract influenced the decrease in tensile strength. Although a decrease in this parameter was observed, the TS was significantly higher than that of other authors for similar studies [44,45]. This is a result of the change in intermolecular density. The addition of the extract, regardless of how it was obtained, caused a change in the distribution of pectin in the biopolymer network, thus the observed decrease in tensile strength (TS).

Incorporation of pectin film matrix extracts resulted in increased plasticity and flexibility of the film. There was also an observed decrease in its strength. Our studies showed that the highest concentrations of polyphenolic compounds were obtained for ethanol extracts. In the case of our films, this was not significant. There was no correlation between the number of polyphenols in the extracts and the change in TS and EB parameters. Therefore, for mechanical strength, the method of extract preparation and the polyphenol content of the extracts we used to obtain the films were not significant.

### 3.5. Optical Tests of Eco-Friendly Packaging Materials

The optical properties of the resulting films (transparency, transmittance, and haze) were also assessed as part of the study, as the color and appearance of packaging materials directly affect consumer acceptability of packaged products and thus the marketing function of the materials. Moreover, optical parameters are functional characteristics that determine information about the size of particles dissolved in the solutions forming the polymers, so that granules and particles larger than visible wavelengths impede the passage of light and increase the opacity of the film produced [46]. In the present study, we used extracts, prepared by different methods, that were rich in active substances that have the potential to interact with biopolymers, resulting in a wide variety of color changes in the produced films [47].

**Figure 11:**
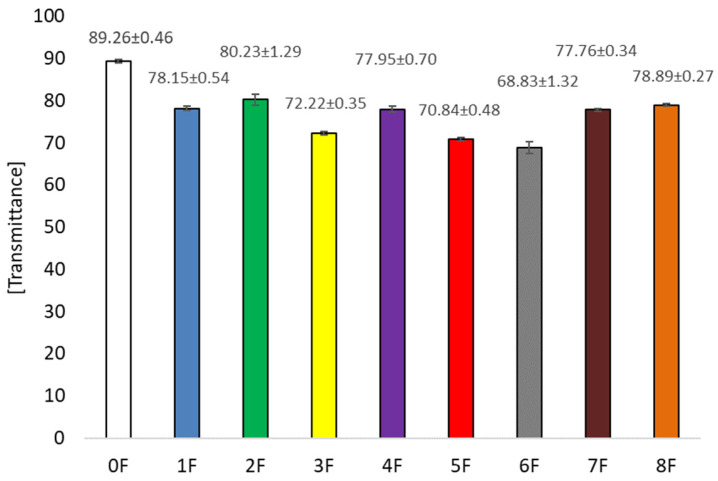
Transmittance of eco-friendly active packaging materials.

**Figure 12:**
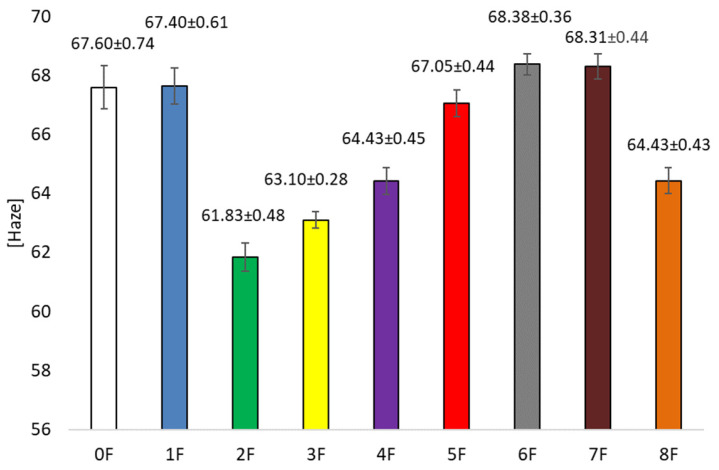
Haze of eco-friendly active packaging materials.

**Figure 13:**
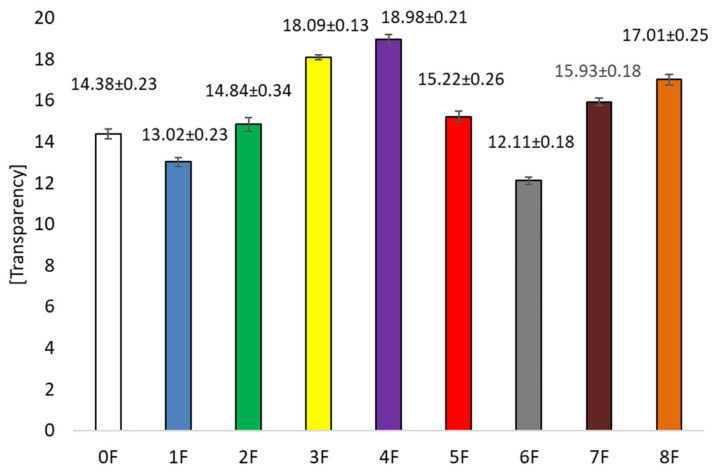
Transparency of eco-friendly active packaging materials.

The highest transmittance value (Figure 11) was recorded for films without extract addition (0F), T = 89.26 ± 0.46 [%]. The addition of extracts caused a decrease in this parameter. The lowest values were for samples 5 and 6, where T was 70.84 ± 0.48 [%] (5F) and 68.83 ± 1.32 [%] (6F), as appropriate. These samples were prepared using ethanol extracts. This study showed that the highest concentrations of polyphenolic compounds were achieved when extracted with a 50% ethanol solution. Consequently, the higher the concentration of polyphenols in the extract used, the lower the transmittance of the films obtained. In the case of haze (Figure 12), H = 67.60 ± 36.53 [%] was observed for the pure film sample. The addition of excipient had no significant statistical effect on the differences between the samples for this parameter. The samples had similar values to sample 0. No effect of the type of extract on the haze of the samples was observed in this case. Statistical tests for Spearman’s rank correlation showed that total polyphenols are inversely proportional to transmittance (R= −0.8333) and migration in Tenax (R= −0.6327). Therefore, the higher the polyphenol content, the lower the transmittance and migration in Tenax. For the transparency test (Figure 13), it was observed that the addition of extracts also influenced the value of this parameter. The pure film without extracts had a value of 14.42 ± 36.53 [%]. The addition of extracts caused an increase in opacity. Films 3 and 4 were characterized by the highest value of the parameter, which were, respectively: 18.14 ± 27.02 and 18.97 ± 29.83 [%]. This is a result of the addition of polyphenolic compound-containing extracts to the biopolymer films, which usually results in increased opacity induced by the formation of cross-links, leading to a change in refractive index and reduced light transmission [48,49]. On the other hand, there are studies suggesting that the reduction in transparency of the films may be due to the rough surface structure, which favors light scattering [15]. In the present study, it was observed that opacity and transparency are inversely proportional [50]. However, higher opacity may be expected when protecting light-sensitive products [51].

### 3.6. Color Testing of Eco-Friendly Packaging Materials

**Figure 14:**
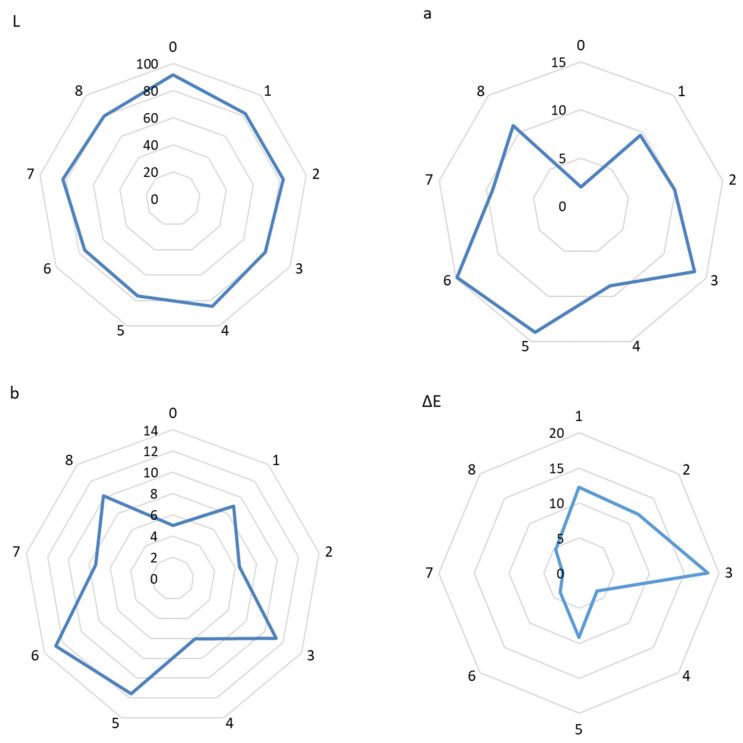
Color testing of eco-friendly active packaging materials.

Figure 14 shows the results of the color test of eco-friendly active packaging materials. The study evaluated the parameters of films, i.e., L* (brightness), a* (red share), b* (yellow share), and the total color difference (ΔE). It was concluded from the evaluation that the inclusion of extracts changed the L*, a*, b*, and color difference (ΔE) values of the packaging materials obtained. Higher values of L indicate greater brightness, while lower values indicate greater darkness. In the present study, a significant decrease in the value of the L parameter was observed (*p* < 0.05). Sample 0 was characterized by a parameter value of L = 91.45. The other films were characterized by a decrease in brightness, which is a result of the addition of the extract to the polymer matrix. Similar results were reported by Chen et al. [52], who observed a decrease in brightness when olive fruit extracts were added to pectin films. In the present study, it was also observed that the decrease in brightness did not depend on the method of obtaining the extract used. There was also a significant decrease in ΔE (*p* < 0.05) for extract-containing films. Also, Biratu et al. (2024) observed a significant change in ΔE after the addition of active substances to pectin matrices. In the case of parameter a* (proportion of red), a definite increase in this parameter was observed (*p* < 0.05), which is the result of the inclusion of colored extracts in the polymer matrix. The film without added extract had a* value of (1.95). In contrast, samples containing extracts with the highest polyphenol content (samples 5 and 6) were characterized by a* values of 13.97 and 14.89, respectively. The b* value (proportion of yellow) for the films also decreased. The lowest b* values were also recorded for samples 5 (11.54) and 6 (12.73). Based on this, it can be concluded that the color of our films varied depending on the method of extraction, with the extract containing the highest values of polyphenols producing the greatest color changes. The results for the color tests (L*, a*, b*, (ΔE)) are confirmed by stereoscopic microscopy, which clearly shows the color of the test samples [53].

### 3.7. Studies on the Migration of Eco-Friendly Packaging Materials

**Figure 15:**
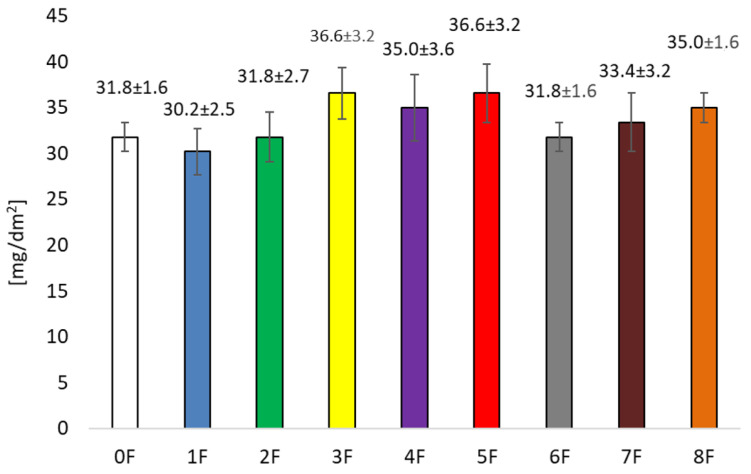
Determination of overall migration into the simulant of neutral food–10% ethanol at a temperature of 40 °C for 10 days.

**Figure 16:**
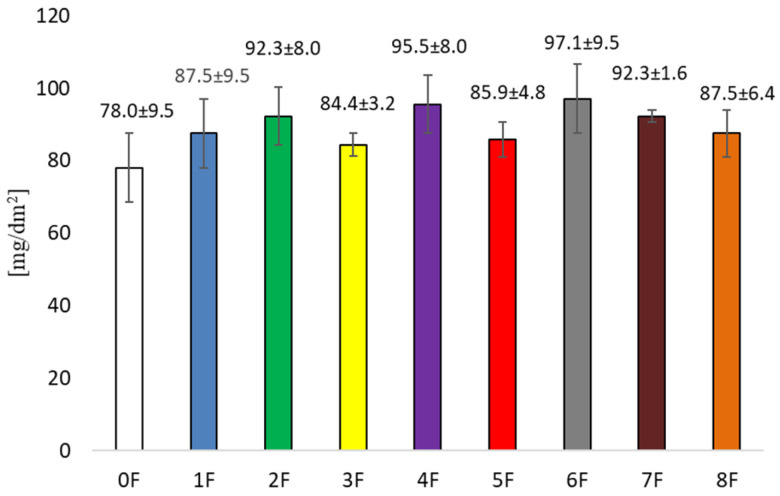
Determination of overall migration into the simulant of volatile alternatives for fatty food–95% ethanol at a temperature of 40 °C for 10 days.

**Figure 17:**
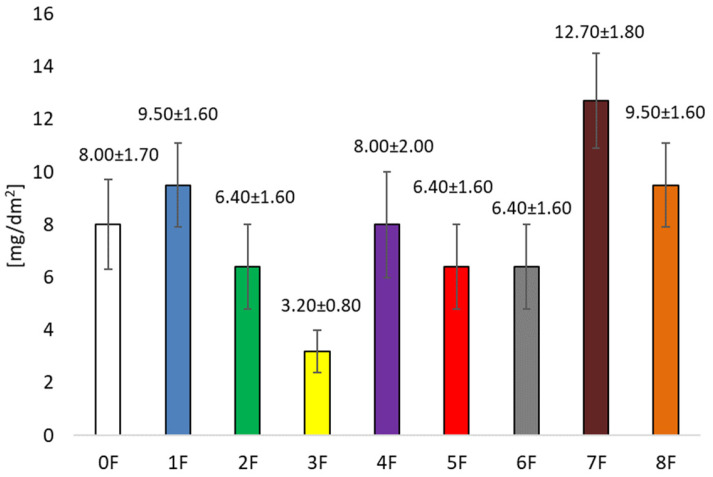
Determination of overall migration into the simulant of volatile alternatives for fatty food–Tenax®, at a temperature of 40 °C for 10 days.

The differences in the amounts of migrated substances from the tested films to the 10% ethanol, 95% ethanol, and Tenax^®^ are most likely caused by the polarity and volatility of the substances present in the films, their nature and the structure of the films (Figure 15, Figure 16 and Figure 17). The lowest migration occurred in the Tenax^®^ (from 3.2 ± 0.8 mg/dm^2^ for film 3F to 12.7 ± 1.8 mg/dm^2^ for film 7F), because it is a solid sorbent. The level of migration was significantly more influenced by volatility and affinity of migrated substances to sorption into solid material. For a liquid material, the highest migration level was determined in 95% ethanol (from 78.0 ± 9.5 mg/dm^2^ for film 0F to 97.1 ± 9.5 mg/dm^2^ for film 6F). In the case of the migration test in 95% ethanol, in all cases of the analysed films, more substances were released than in the migration test of the blank sample (film 0F). This indicates the presence of substances in added extracts with a tendency for migration into ethanol. This fact was strengthened by results for migration into 10% ethanol, where the migration level was more than twice lower than for 95% ethanol. At the same time, the achievement of a higher migration value to 95% ethanol is limited by the total extract content in the film. Achieving a lower level of migration to 10% ethanol is limited by the ability of pectin to partially dissolve in water. However, the migration results to 10% ethanol (from 32.2 mg/dm^2^ for film 1F to 36.2 mg/dm^2^ for film 5F) show that even these results exceed the overall migration limit of 10 mg/dm^2^ set by Regulation 10/2011. The results also indicate the limited inertness of these materials to liquid foods. This could be problematic for the health and safety assessment of packaging materials consisting of these films, especially in the case of the components of the extracts added to the film, because the release of the naturally occurring pectin in the food itself is not a problem. Migration to Tenax^®^ complies with the limit of 10 mg/dm^2^ except film 7F.

### 3.8. Images of Films of Eco-Friendly Packaging Materials

**Figure 18:**
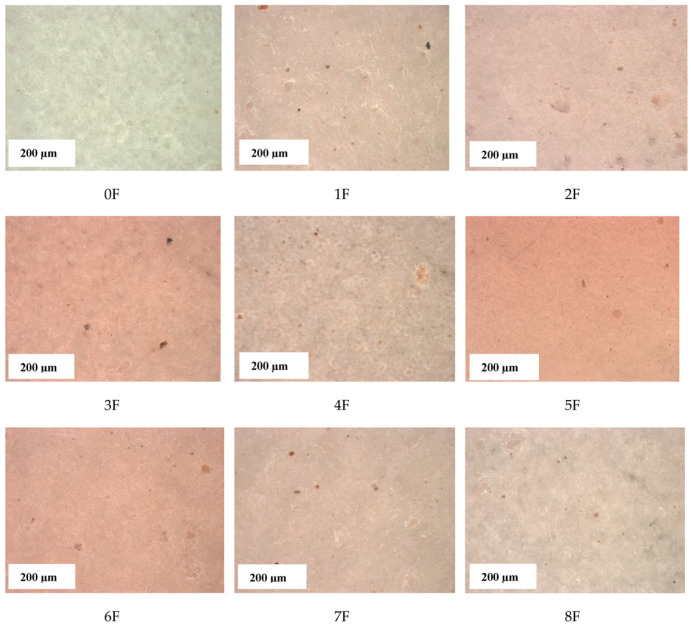
Stereoscopic images for samples 0–8 (scale 200 µm).

**Figure 19:**
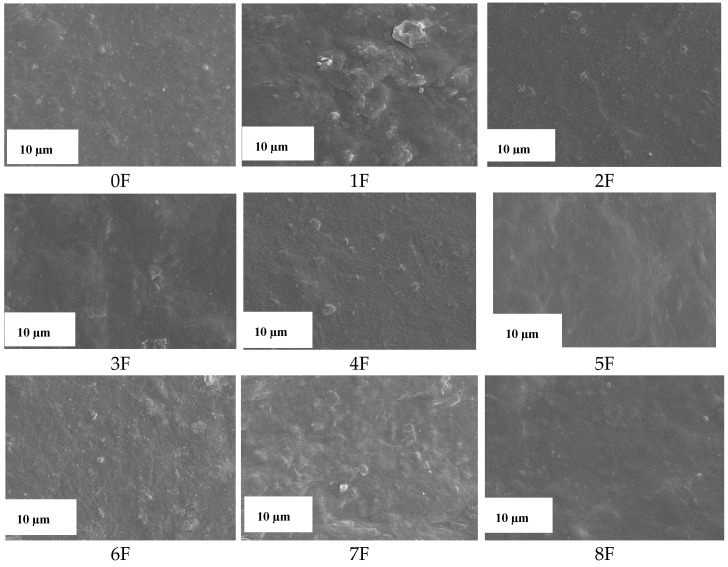
Scanning images for samples 0–8 (scale 10 µm).

**Figure 20:**
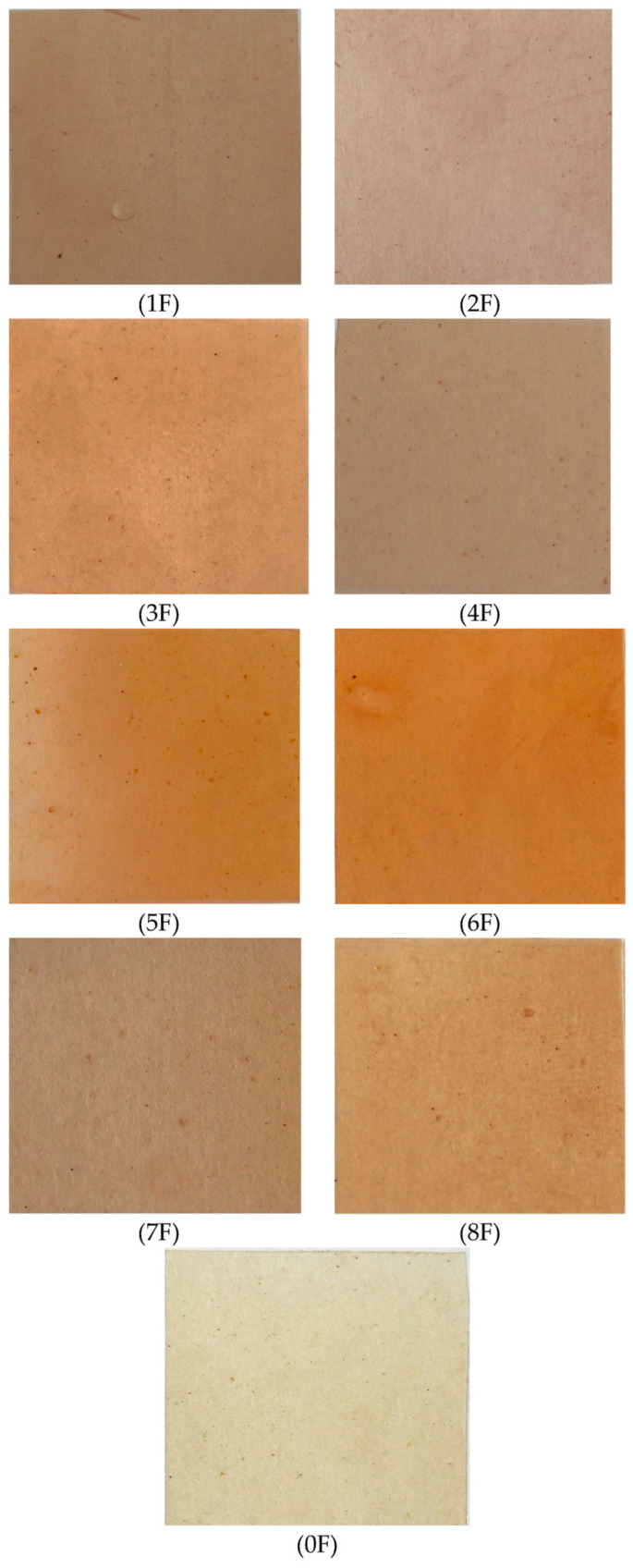
Photos of finished films.

In the present study, the morphology and distribution of the extract in the film matrix were assessed using a scanning electron microscope and a stereoscopic microscope. The analysis of the sample surface allowed a better understanding of the mechanical properties and barrier mechanisms. Figure 18 and Figure 19 present scanning and stereoscopic images of eco-friendly active packaging materials with green extracts from fruit processing waste. The SEM images taken for the samples show homogeneous surfaces with minor irregularities in some regions. The images show a smooth and homogeneous surface with small, dispersed particles, which can be attributed to the incomplete dissolution of the polysaccharides used. For all films, independent of concentration, the surface was smooth and showed no roughness. Furthermore, no breaks or phase separation were observed on the surfaces of the films, suggesting a coherent matrix. Moreover, the tested samples showed a continuous microstructure. The structure of the resulting film is closely related to the interaction between its components. This is evidence of a strong interaction between PA, TG, and the extracts used. This, in turn, has far-reaching consequences for its different properties. Photographs taken with a stereoscopic microscope confirm the surface smoothness evidenced by the SEM images and, additionally, show a clear change in the color of the film depending on the type of extract used. These results complement the optical tests carried out previously and the color analysis of the samples obtained. Figure 20 shows images of the finished films.

### 3.9. Statistical Analysis of the Results Obtained

#### 3.9.1. Spearman’s Rank Correlation

Table 2 shows the correlation coefficients between the parameters determined for each film.

The parameters of total polyphenols and antioxidant activity were found to be almost completely correlated (0.9 ≤ R < 1). This correlation has already been found in earlier studies by many researchers, as polyphenolic compounds exhibit high antioxidant activity. The parameters: oxygen permeability and water vapor transmission rate; antioxidants TEAC and overall migration Tenax; overall migration 10% ethanol and transparency; transmittance and total polyphenols; and transmittance and antioxidants TEAC were found to be highly correlated (0.7 ≤ R < 1). Also highly correlated (0.5 ≤ R < 0.7) were the parameters: water vapor transmission rate and overall migration Tenax; oxygen permeability and overall migration 10% ethanol; oxygen permeability and haze; overall migration Tenax and total polyphenols; elongation at break and overall migration Tenax; and haze and transparency. Figure 20 presents the results of the cluster analysis.

#### 3.9.2. Cluster Analysis

**Figure 21:**
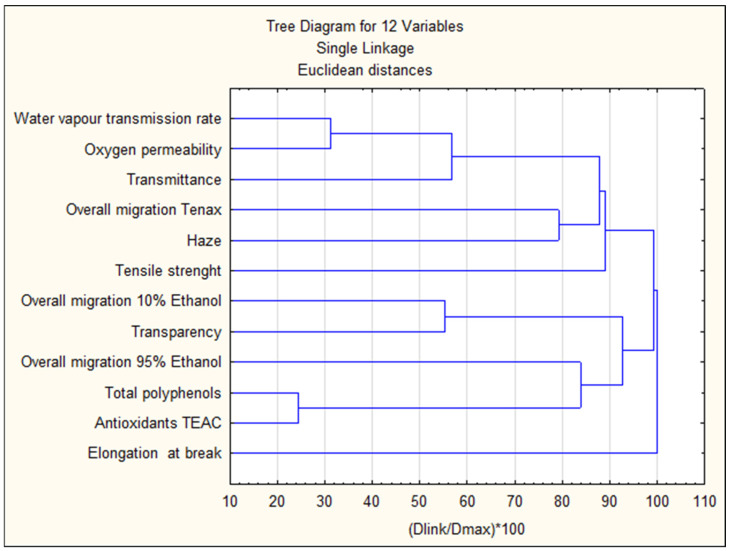
Results of the cluster analysis.

Figure 21 shows the results of the cluster analysis.

Cluster analysis showed that total polyphenol content and antioxidant activity are the most closely related, these parameters forming direct and least-distant relationships. These parameters are further related to the results of overall migration in 95% ethanol. The analysis also found that related to each other and forming direct and least distant linkages were the parameters water vapor transmission rate and oxygen permeability, followed by a linkage with transmittance. Direct links were also found between overall migration in 10% ethanol and transparency and migration in Tenax with haze. The parameter elongation at break is linked to all parameters. These correlations are not necessarily reflected in the values of the rank correlation coefficients, as the R-score for ranks only characterizes linear relationships, while the effects under study may be higher-degree relationships.

## 4. Conclusions

In this research, an attempt was made to design environmentally friendly active packaging materials with green extracts from fruit processing waste that fit into a closed-loop economy. The study showed that qualitative and quantitative changes in the profile of polyphenolic compounds are affected by the extraction parameters used. Films containing ethanolic extracts showed significantly higher amounts of polyphenolic compounds compared to those with aqueous extracts, with the highest content in the film containing the extract achieved by shaking at 60 °C. The type of extract used also influenced the mechanical, barrier, and optical properties of the film. Cluster analysis showed that total polyphenol content and antioxidant activity are most closely related. These parameters are correlated with the overall migration results in 95% ethanol. The analysis also showed that water vapor permeation rate and oxygen permeability parameters were correlated and formed direct and least-distant relationships. Thus, the research achieved its main objective, i.e., materials with bioactive potential, allowing the quality of the packaged product to be improved, while taking care of ecology and sustainability.

## Figures and Tables

**Table 1:** Extraction variants.

Sample	Conditions	Extraction Solvent
1 (E/F)	Ultrasonic bath (80 W, 0.5 h)	ethanol 50%
2 (E/F)	Ultrasonic bath (80 W, 0.5 h)	water
3 (E/F)	Ultrasonic bath (80 W, 2 h)	ethanol 50%
4 (E/F)	Ultrasonic bath (80 W, 2 h)	water
5 (E/F)	Shaking (130 rpm, 25 °C, 3 h)	ethanol 50%
6 (E/F)	Shaking (130 rpm, 60 °C, 3 h)	ethanol 50%
7 (E/F)	Shaking (130 rpm, 25 °C, 3 h)	water
8 (E/F)	Shaking (130 rpm, 60 °C, 3 h)	water

Symbols E or F relate to variants of extracts (E) or films (F).

**Table 2:** The correlation coefficients between film properties.

	Water Vapor Transmission Rate	Oxygen Permeability	Overall Migration 10% Ethanol	Overall Migration 95% Ethanol	Overall Migration Tenax	Total Polyphenols	Antioxidants Teac	Elongation at Break	Tensile Strength	Haze	Transmittance	Transparency
Water vapor transmission rate	1.0000	0.7167	−0.3334	−0.0504	0.5301	−0.4000	−0.4667	−0.2167	−0.1333	0.3833	0.3500	−0.1000
Oxygen permeability	0.7167	1.0000	−0.5814	−0.1429	0.2650	−0.2000	−0.2833	0.0833	−0.1500	0.5000	0.3000	−0.6667
Overall migration 10% Ethanol	−0.3334	−0.5814	1.0000	−0.2543	−0.3421	0.1881	0.3163	−0.3163	0.3591	−0.4275	−0.4189	0.7780
Overall migration 95% Ethanol	−0.0504	−0.1429	−0.2543	1.0000	0.1380	0.2605	0.3698	0.0504	−0.3361	0.2185	−0.3109	−0.0756
Overall migration Tenax	0.5301	0.2650	−0.3421	0.1380	1.0000	−0.6327	−0.7096	0.5130	−0.2565	0.4446	0.3676	0.0085
Total polyphenols	−0.4000	−0.2000	0.1881	0.2605	−0.6327	1.0000	0.9333	−0.1333	−0.3167	0.1000	−0.8333	−0.1500
Antioxidants TEAC	−0.4667	−0.2833	0.3163	0.3698	−0.7096	0.9333	1.0000	−0.2833	−0.0500	−0.0167	−0.8333	−0.0500
Elongation at break	−0.2167	0.0833	−0.3163	0.0504	0.5130	−0.1333	−0.2833	1.0000	−0.4667	0.2333	0.1167	−0.2833
Tensile strength	−0.1333	−0.1500	0.3591	−0.3361	−0.2565	−0.3167	−0.0500	−0.4667	1.0000	−0.3667	0.2000	0.2167
Haze	0.3833	0.5000	−0.4275	0.2185	0.4446	0.1000	−0.0167	0.2333	−0.3667	1.0000	−0.3500	−0.6000
Transmittance	0.3500	0.3000	−0.4189	−0.3109	0.3676	−0.8333	−0.8333	0.1167	0.2000	−0.3500	1.0000	−0.0167
Transparency	−0.1000	−0.6667	0.7780	−0.0756	0.0085	−0.1500	−0.0500	−0.2833	0.2167	−0.6000	−0.0167	1.0000

Spearman Rank Order Correlations, MD pairwise deleted Marked correlations are significant at *p* < 0.05.

## Data Availability

The original contributions presented in the study are included in the article, further inquiries can be directed to the corresponding author.
